# Valproic acid influences the expression of genes implicated with hyperglycaemia-induced complement and coagulation pathways

**DOI:** 10.1038/s41598-021-81794-4

**Published:** 2021-01-25

**Authors:** Marina Barreto Felisbino, Mark Ziemann, Ishant Khurana, Jun Okabe, Keith Al-Hasani, Scott Maxwell, K. N. Harikrishnan, Camila Borges Martins de Oliveira, Maria Luiza S. Mello, Assam El-Osta

**Affiliations:** 1 Department of Structural and Functional Biology, Institute of Biology, University of Campinas (Unicamp), Campinas, SP 13083-862 Brazil; 2 Epigenetics in Human Health and Disease Laboratory, Department of Diabetes, Central Clinical School, The Alfred Medical Research and Education Precinct, Monash University, Melbourne, VIC Australia; 3 Department of Medicine and Therapeutics, The Chinese University of Hong Kong, Hong Kong SAR, China; 4 Hong Kong Institute of Diabetes and Obesity, Prince of Wales Hospital, The Chinese University of Hong Kong, 3/F Lui Che Woo Clinical Sciences Building, 30-32 Ngan Shing Street, Sha Tin, Hong Kong SAR, China; 5 Li Ka Shing Institute of Health Sciences, The Chinese University of Hong Kong, Hong Kong SAR, China; 6 Faculty of Health, Department of Technology, Biomedical Laboratory Science, University College Copenhagen, Copenhagen, Denmark

**Keywords:** Endocrine system and metabolic diseases, Hepatology, Diabetes

## Abstract

Because the liver plays a major role in metabolic homeostasis and secretion of clotting factors and inflammatory innate immune proteins, there is interest in understanding the mechanisms of hepatic cell activation under hyperglycaemia and whether this can be attenuated pharmacologically. We have previously shown that hyperglycaemia stimulates major changes in chromatin organization and metabolism in hepatocytes, and that the histone deacetylase inhibitor valproic acid (VPA) is able to reverse some of these metabolic changes. In this study, we have used RNA-sequencing (RNA-seq) to investigate how VPA influences gene expression in hepatocytes. Interesting, we observed that VPA attenuates hyperglycaemia-induced activation of complement and coagulation cascade genes. We also observe that many of the gene activation events coincide with changes to histone acetylation at the promoter of these genes indicating that epigenetic regulation is involved in VPA action.

## Introduction

Diabetes mellitus is a multifactorial disorder with several pathways implicated in the development of diabetic micro- and macro-vascular complications^1^. Macrovascular complications include atherosclerosis, cerebrovascular disease and peripheral vascular disease; for which diabetic patients have a two- to four-fold greater risk than non-diabetic individuals^2^. In diabetes, a combination of hyperglycaemia, inflammation, oxidative stress and insulin resistance converge to produce a prothrombotic milieu, characterised by endothelial dysfunction, coagulative activation and platelet hyperreactivity, reviewed in^3^.

Several important coagulation pathway proteins are elevated by hyperglycaemia in vivo including fibrinogen, prothrombin 1 and 2, tissue factor, thrombin-antithrombin complexes, plasminogen activator inhibitor-1, tissue plasminogen activator and complement C3 which contribute to this hypercoagulative state^4–6^. This prothrombotic state co-exist with chronic low-grade inflammation and oxidative stress^7^. Complement cascade proteins are a proposed source of inflammation in diabetes that are produced by monocytes, macrophages and hepatocytes. These proteins function in innate antimicrobial defense primarily through membrane attack and phagocyte recruitment that are elevated in diabetes and suspected to contribute to diabetic complications^8^. The liver is the main site of production of circulating coagulation and complement proteins, also responsible for production of bile, cholesterol, decomposition of red blood cells and detoxification of xenobiotics and secondary metabolites^9–12^. Of key importance for diabetes, liver hepatocytes play a major role in energy homeostasis by storing carbohydrates as glycogen during hyperglycaemia and releasing sugars during hypoglycemia. Liver dysfunction, classified as elevated hepatic glucose production during hyperglycaemia, is common in type-2 diabetes, and inhibiting this is a major mechanism of action of the widely prescribed glucose lowering drug metformin^13^. Thus, understanding the molecular mechanisms underpinning the hyperglycaemic activation of metabolism, coagulation, complement and other inflammatory pathways in hepatocytes could identify new therapies to reduce the burden of diabetic complications.

At the interface between genetic and environmental factors, epigenetic mechanisms are proposed to play a major role in the development of metabolic disease including diabetic complications^14,15^. Previous reports have demonstrated that chromatin remodeling and histone acetylation are important mechanisms in diabetes development^16,17^. The epigenetic component of metabolic/inflammatory disorders has come recently to attention, revealing epigenetic drugs as potential immunomodulatory agents. The recent discovery that histone deacetylase (HDAC) inhibitors (HDACi) have the ability to reduce the severity of inflammatory and autoimmune diseases, including diabetes, in several animal models, has positioned them as alternative anti-inflammatory agents^18–21^. Their paradigmatic mode of action has been defined as increased histone acetylation of target genes, leading to higher gene expression; however, recent studies have shown a more diverse mechanism of gene regulation^22–25^.

Valproic acid (VPA; IUPAC: 2-propylpentanoic acid), the most clinically prescribed HDACi, is a fatty acid with anticonvulsant properties used for the treatment of epilepsy and seizures^26^. VPA inhibits class I (HDAC1, HDAC2, HDAC3, HDAC8) and class IIa (HDAC4, HDAC5, and HDAC7), leading to an increase in the acetylation of histones H2, H3, and H4, which modify the expression of associated genes^21^. Recently, its use has been investigated associated with different diseases as a strategy of repurposing clinically approved drugs^27–29^. There are reports of VPA reducing the blood glucose level and fat deposition in adipose tissue and liver of mice and rats^30,31^, while Class I^32,33^ and class IIa^34^ HDACs seems to be involved in the control of gluconeogenesis signaling and the insulin production. VPA also reduces the microvascular complications of diabetes^35,36^.

We have previously shown that treatment of HepG2 human hepatocytes with the HDACis Trichostatin A (TSA) and VPA attenuated hepatic glucose production, although no significant difference was detected in global chromatin structure and epigenetic landscape. Chromatin alterations promoted by HDACi under hyperglycaemia may be a function of the differently regulated nuclear domains and genes rather than of global remodeling^17^. Therefore, identification of genes influenced by HDAC inhibition is paramount to understanding of its mechanisms of action and therapeutic target in amelioration of hyperglycaemic state^23^. We hypothesise that hepatocytes undergo major gene expression alterations when exposed to a hyperglycaemic environment as the liver is an organ of critical importance to carbohydrate metabolism. Furthermore, we hypothesised that VPA could attenuate some of the deleterious pathways promoted by hyperglycaemia by conferring changes to promoter histone acetylation.

In this study, HepG2 cells exposed to high-glucose (HG) were stimulated with VPA. We performed high throughput RNA-sequencing (RNA-seq) to understand transcriptome-wide analysis of genes and pathways in response to hyperglycaemia and VPA. We observe genes influenced by VPA are altered in H3K9ac at their promoters. This work identified that complement and coagulation pathways activated by hyperglycaemia were attenuated by HDAC inhibition.

## Results

### Hyperglycaemia regulates hepatocyte gene expression

**Figure 1:**
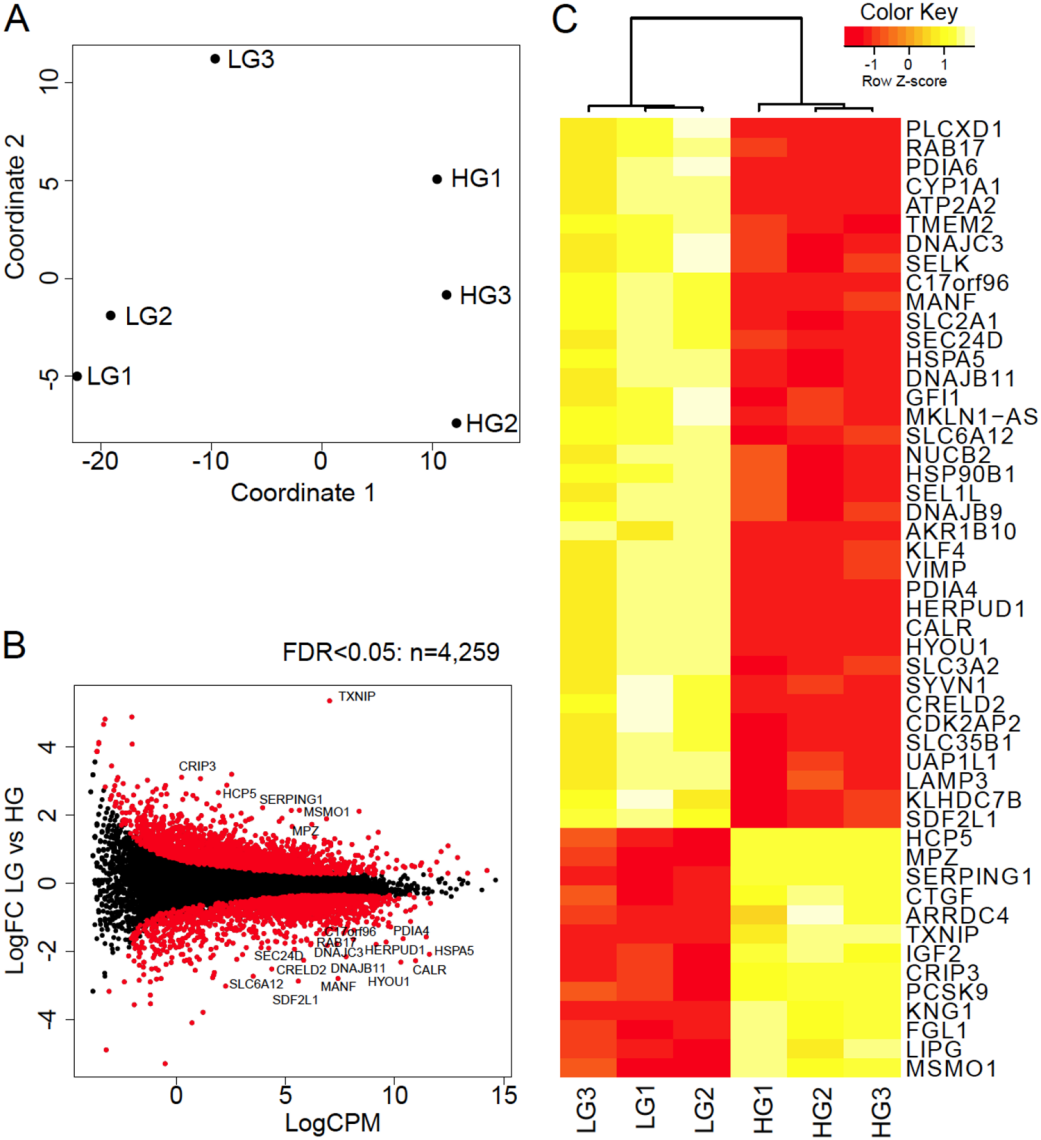
RNA-seq identifies gene expression changes due to high glucose exposure in HepG2 cells. (**A**) Multidimensional scaling analysis shows sample clustering based upon treatment. (**B**) Smear plot depicts the log2 fold change and average expression level log2 counts per million for each detected gene. Genes with differential expression (FDR ≤ 0.05) are highlighted in red. (**C**) Heatmap of the 50 most significant differentially expressed genes. LG, low glucose—normoglycemic condition. HG, high glucose—hyperglycaemic condition.

In order to understand the effect of high glucose on whole genome hepatic gene expression, RNA-seq was performed on HepG2 cells cultured under continuous low glucose (LG) or stimulated with 48 h high glucose (HG; 20 mM) in triplicate. After read alignment and gene expression quantification, differential expression analysis of genes and pathways was undertaken. Multidimensional scaling analysis measures the similarity of the samples and projects this on two-dimensions. We observed that LG and HG samples clustered into distinct groups (Fig. 1A). Statistical analysis showed that HG treatment had a strong effect on HepG2 cells, with 4259 genes (26%) showing differential expression (FDR ≤ 0.05; Fig. 1B—red points). This effect in gene expression is greater than that reported previously for high glucose treated THP-1 human monocytic cell line^37^ and skeletal muscle of diabetic Goto–Kakizaki when compared to control Wistar rat^38^ suggesting that hepatic cells are especially sensitive to alterations in glucose level. The top 50 differentially expressed genes by significance are shown in heatmap form (Fig. 1C). Some of the genes influenced by HG are also highlighted in Table 1.

**Table 1:** Selected genes differently regulated by hyperglycaemia exposure in HepG2 cells.

Gene	Function	Log2 fold change	FDR
*TXNIP*	A known hyperglycaemia inducible gene that is highly abundant in HepG2 cells. TXNIP protein inhibits the normal function of thioredoxin leading to accumulation of ROS	5.38	2.99E−45
*SERPING1*	Encodes C1 esterase inhibitor and is involved in inhibition of complement cascade	2.17	6.72E−45
*MPZ*	Encodes a structural component of the myelin sheath and is thought to be specific to nervous system tissues	1.70	2.61E−29
*MSMO1*	Encodes a protein involved in cholesterol biosynthesis	1.76	2.09E−25
*PCSK9*	Protein acts binding to and degrading low-density lipid receptors	1.93	4.85E−20
*IGF2*	Encodes an insulin-like growth factor, the central regulator of somatic growth and cell proliferation	1.13	4.81E−18
*MANF*	Protein promotes survival of dopaminergic neurons, possibly playing a role in ER stress response	− 2.75	3.41E−64
*HSPA5* (aka *GRP78*)	Encodes a glucose sensing protein, whose expression is upregulated by glucose starvation and is also present in the ER	− 2.04	1.93E−45
*CALR*	Encodes calreticulin, a major calcium storage protein in the ER	− 2.23	9.49E−40
*DNAJB11*	Encodes a ER localized protein that acts as a chaperone for a number of partners	− 2.12	1.65E−36
*SLC2A1*	The glucose transporter gene that encoding GLUT1	− 1.19	1.20E−20

FDR, false discovery rate.

**Figure 2:**
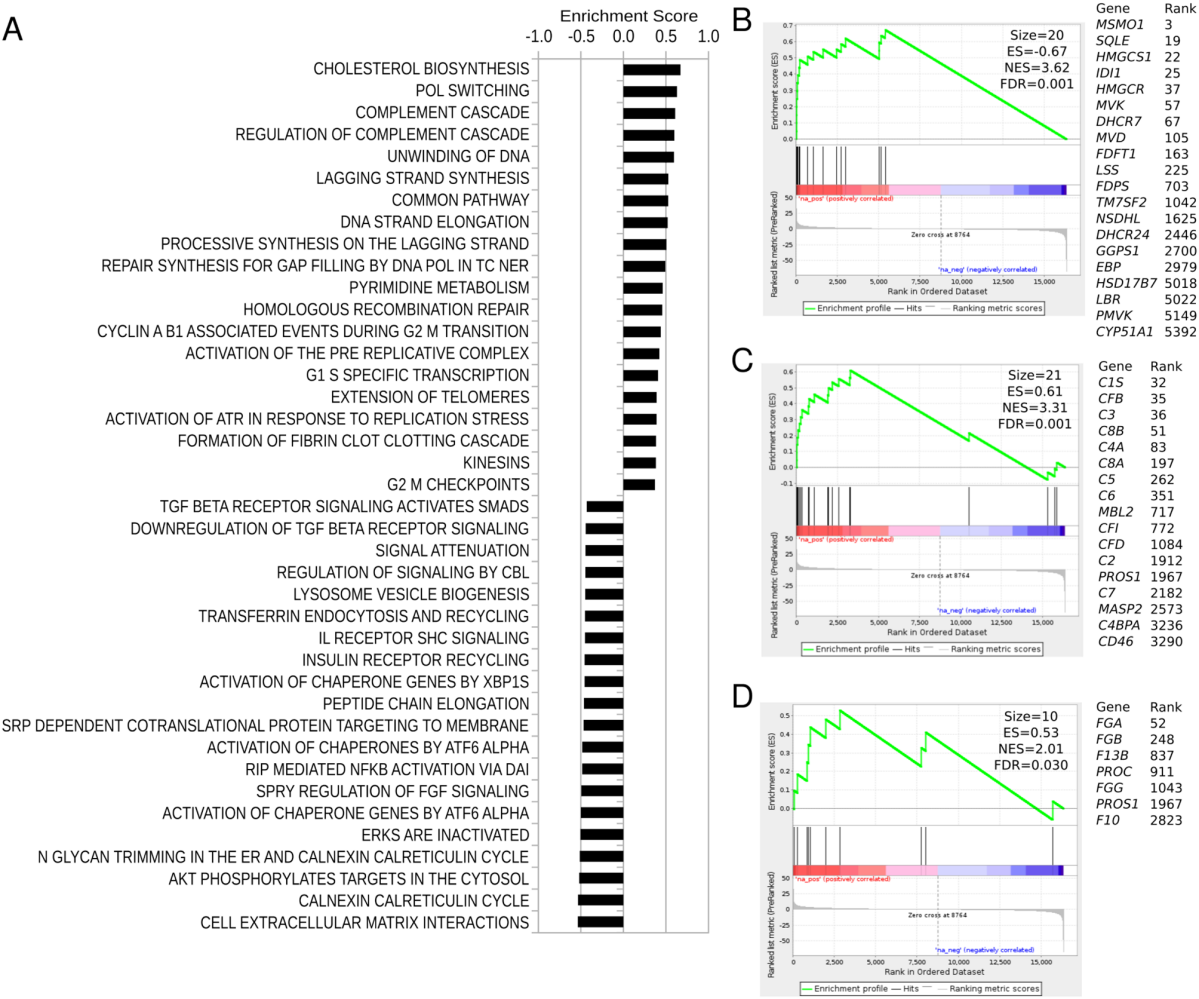
GSEA identifies differentially expressed REACTOME pathways in response to high glucose. (**A**) Top 20 up- and down-regulated pathways selected by statistical significance. (**B**–**D**) Enrichment plots show upregulation of cholesterol biosynthesis, complement cascade and common clotting cascade in response to hyperglycaemia. Beside each enrichment plot, we have included a list of genes that contribute to the enrichment (“leading edge”). GSEA FDR < 0.05 for all pathways shown.

Gene Set Enrichment Analysis (GSEA) was used in order to understand pathways regulated by hyperglycaemia. From 575 REACTOME gene sets considered, 34 were upregulated and 139 were down-regulated (FDR ≤ 0.05). The top 20 gene sets by significance in the up and down-regulated directions are shown (Fig. 2A). Down-regulated gene sets included those associated with extracellular matrix interactions, chaperone function, calnexin/calreticulin cycle, N-glycan trimming and peptide chain elongation (Fig. 2A), while gene sets upregulated in response to hyperglycaemia included cholesterol biosynthesis, complement cascade and fibrin clotting cascade (Fig. 2B–D). These findings show a distinctive severe response of hepatocytes to hyperglycaemia.

### VPA treatment influences the expression of hyperglycaemic response genes

**Figure 3:**
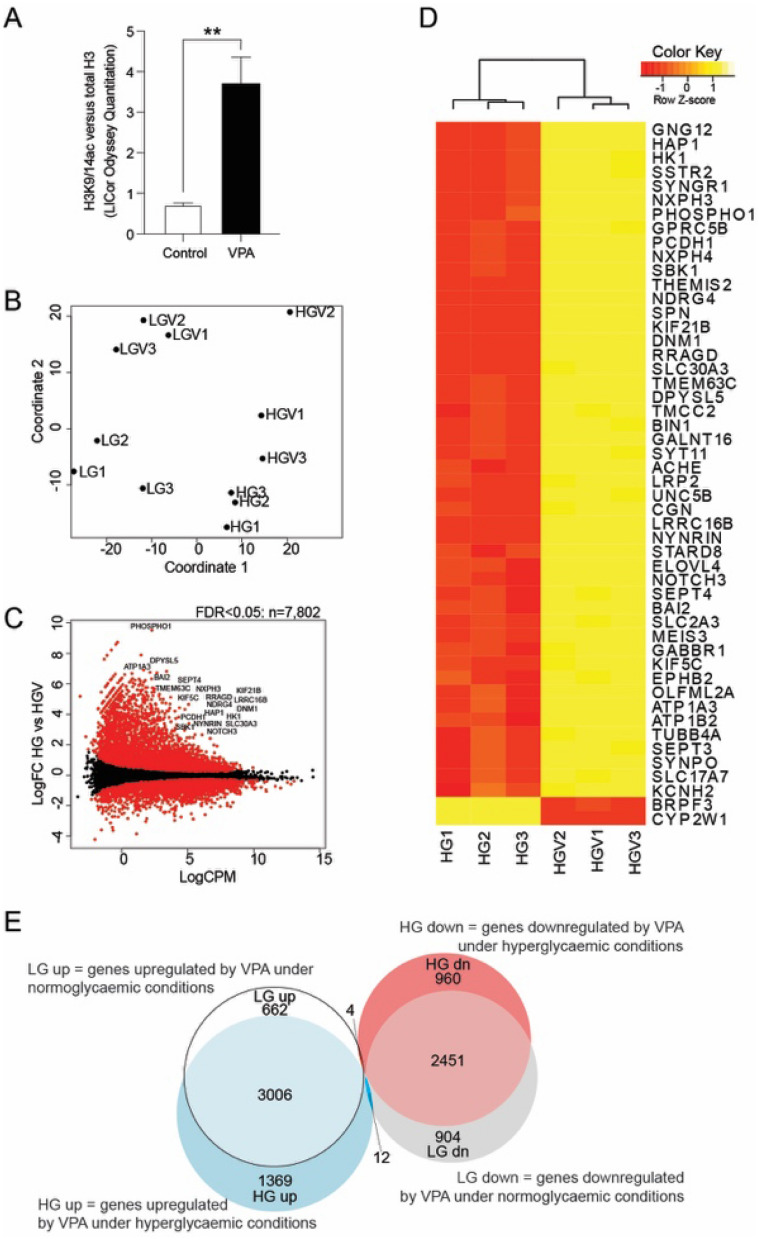
Effect of VPA stimulation on hyperglycaemic HepG2 gene expression. (**A**) VPA induces H3 acetylation in a time-dependent manner. Quantitative analysis of H3K9/14ac and unmodified histone H3 protein using LI-COR Odyssey imaging system. Normoglycaemic HepG2 cells treated with 1 mM VPA in comparison with untreated control. (**B**) Multidimensional scaling analysis shows samples cluster based upon treatment groups. (**C**) Smear plot showing the effect of VPA treatment on hyperglycaemic HepG2 cells. Genes with differential expression (FDR ≤ 0.05) are highlighted in red. (**D**) Heatmap of the 50 most significant differentially expressed genes responding to VPA. LG, low glucose—normoglycemic condition. LGV, normoglycemic condition followed by VPA treatment. HG, high glucose—hyperglycaemic condition. HGV, hyperglycaemic condition followed by VPA treatment. (**E**) Venn diagram showing gene expression changes regulated by VPA under normoglycaemic and hyperglycaemic conditions.

Given that hyperglycaemia induces changes to the hepatocyte transcriptome and activates pathways relevant to cardiovascular health (such as cholesterol metabolism and complement/clotting cascades) and our previous work shows that VPA attenuates hepatic function, we hypothesised that VPA might inhibit hyperglycaemic gene expression signatures. To resolve this, cells under LG and HG conditions for 48 h were exposed to 1.0 mM VPA for a further 12 h. The gene expression profiles were compared to the respective controls without VPA. Quantitative analysis of histone H3K9/14ac protein using LI-COR Odyssey imaging system show significant hyperacetylation in response to VPA (Fig. 3A).

Multidimensional scaling analysis shows that samples cluster based on treatment group. Untreated samples (LG, HG) are clearly separated from VPA-treated samples (LGV, HGV); and normoglycemic samples (LG, LGV) are separated from hyperglycaemic ones (HG, HGV) (Fig. 3B). Smear plot shows that 7802 genes were altered in expression due to VPA treatment under hyperglycaemia (Fig. 3C). This plot also shows genes with initially low expression were upregulated after VPA treatment; on the other hand, genes initially highly expressed were down-regulated. Heatmap of top 50 genes by significance shows that the majority were upregulated (Fig. 3D) and differential gene expression modulated by VPA under normoglycaemic and hyperglycaemic conditions (Fig. 3E).

**Figure 4:**
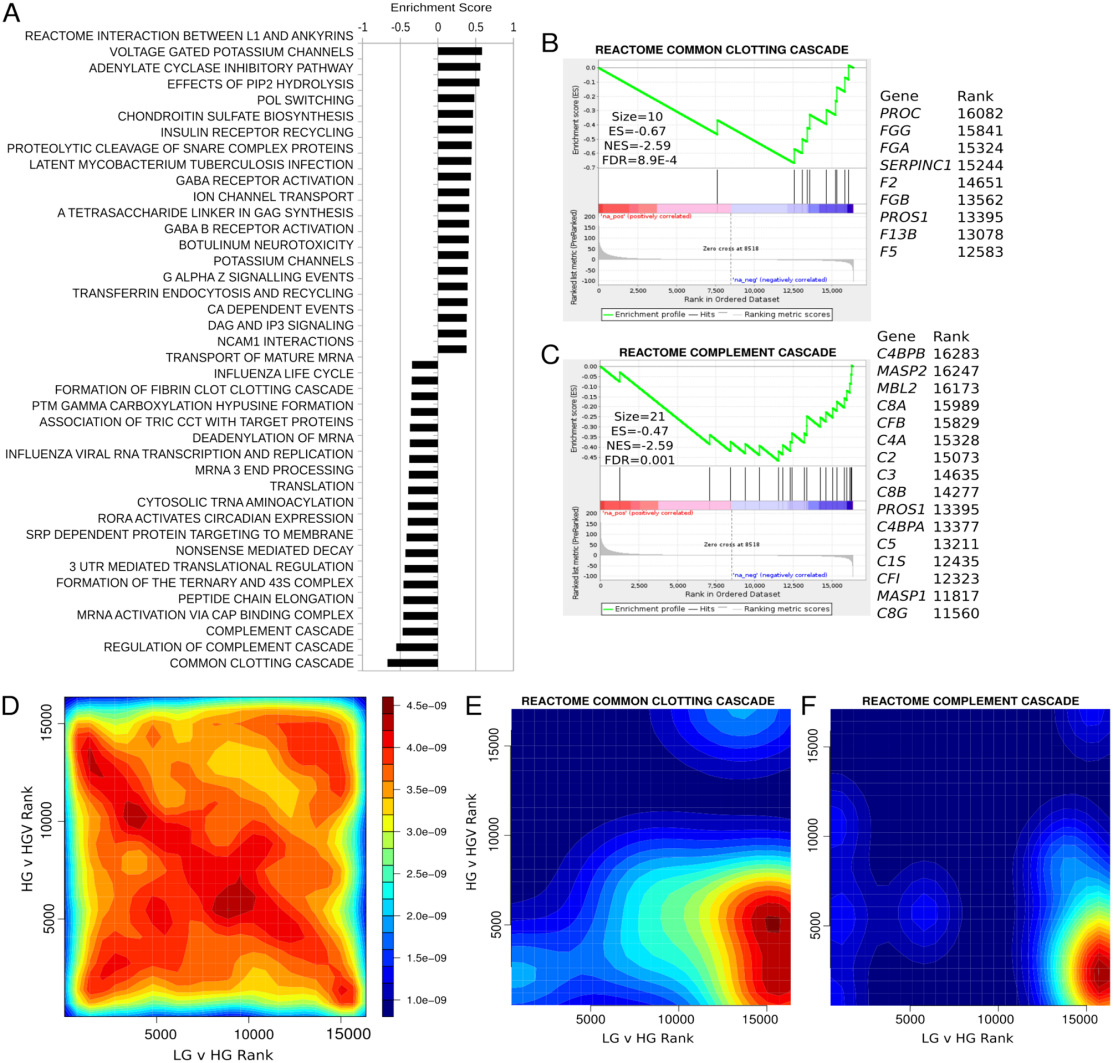
VPA attenuates expression of clotting and complement pathways in hyperglycaemic HepG2 cells. (**A**) Top 20 REACTOME pathways with differential expression in response to VPA under hyperglycaemia determined with ranked gene set enrichment analysis (GSEA-P, FDR < 0.05). (**B**,**C**) Enrichment plot shows downregulation of common clotting and complement cascades in response to VPA. (**D**) Rank-rank plots of gene expression changes for all detected genes and (**E**,**F**) specifically for genes in the common clotting and complement cascade pathways.

Next, we sought to identify gene sets altered by VPA in the context of high glucose. The top 20 gene sets by significance in the up- and down-regulated directions are shown (Fig. 4A). Gene sets upregulated included those related to function of neurons including potassium channels, neurotransmitter receptor, L1-type/ankyrins interactions. Down-regulated gene sets included common pathway of fibrin clot formation, complement cascade and genes involved in protein synthesis. Clotting and complement cascade genes were down-regulated by VPA in hyperglycaemic condition (Fig. 4B,C). The regulation of all genes in response to glucose and VPA was visualised on a two dimensional rank–rank plot (Fig. 4D). We observe that overall, genes are distributed relatively evenly among the four quadrants. Using rank–rank visualisation of clotting and complement cascade genes we observed coordinated upregulation of these genes with hyperglycaemia and attenuation by VPA (Fig. 4E,F). The FDR corrected MANOVA *p* values for the two-dimensional association were 2.0E−4 and 1.5E−7 for clotting and complement cascades respectively.

**Figure 5:**
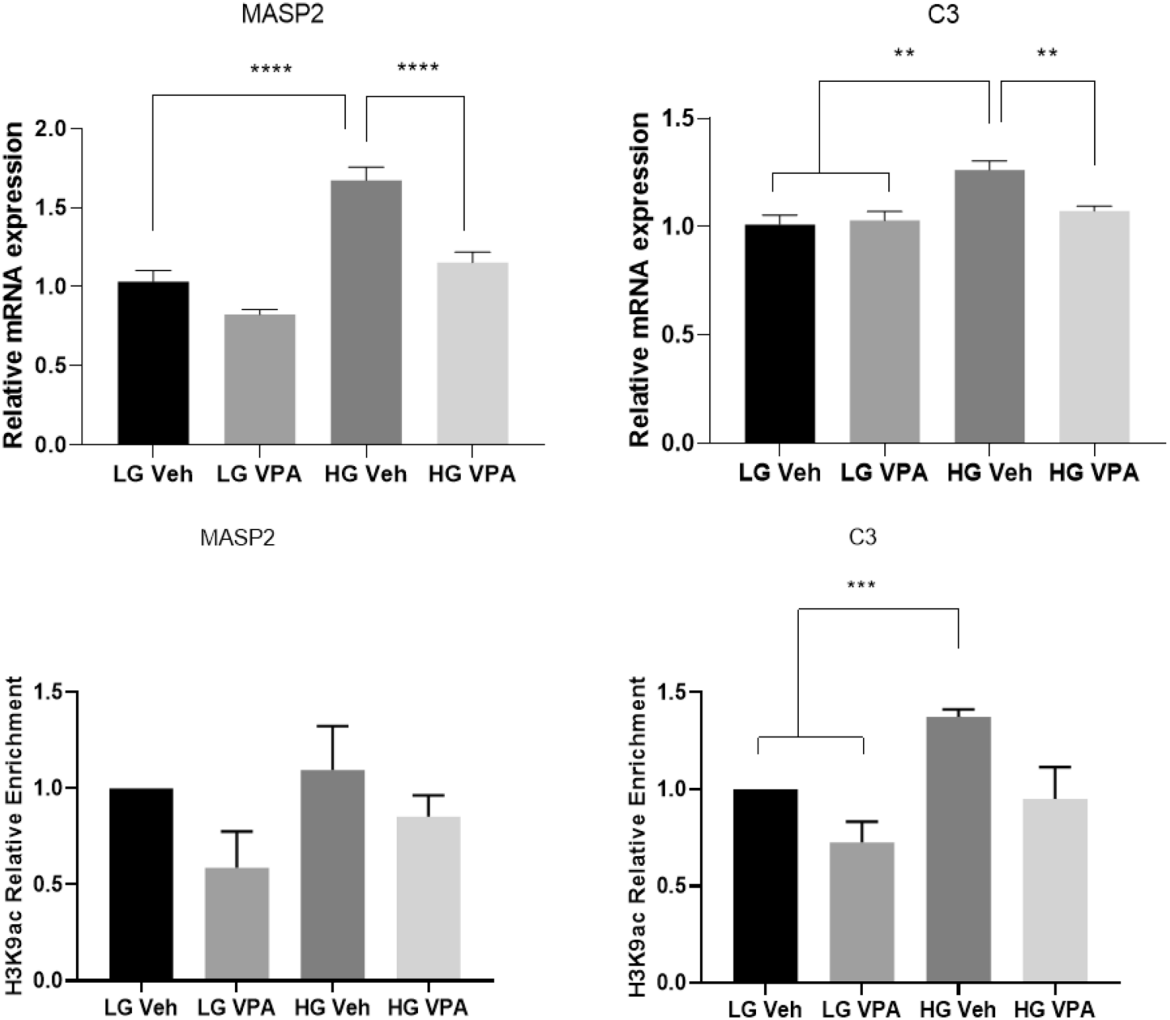
Validation of complement pathway gene (*MASP2*, *C3*) RNA expression and promoter H3K9ac enrichment. VPA stimulation attenuates the expression of these genes and is consistent with changes in promoter H3K9ac. LG, low glucose—normoglycemic condition. LGV, normoglycemic condition followed by VPA treatment. HG, high glucose—hyperglycaemic condition. HGV, hyperglycaemic condition followed by VPA treatment. *p < 0.05; **p < 0.01; ***p < 0.001 as determined by one-way anova analysis.

To validate some of the differentially expressed genes from the RNA-seq findings, we cultivated HepG2 cells under hyperglycaemic conditions prior to treatment with VPA as above described followed by quantitative reverse transcription PCR (RT-qPCR). The selected genes included those involved in the complement (*MASP2* and *C3*) pathway. Selected genes upregulated by hyperglycaemia according to the RNA-seq results, and confirmed by real-time PCR, were attenuated by VPA stimulation in agreement with RNA-seq (Fig. 5). As proof of concept, we assessed the relative abundance of H3K9ac at the promoter of the *MASP2* and *C3* genes using chromatin immunoprecipitation (ChIP) and qPCR detection. We observe reduced H3K9ac in VPA treated cells and under hyperglycaemic conditions H3K9ac is partly attenuated suggesting hyperglycaemia-induced expression of complement and clotting genes could be regulated by histone acetylation.

## Discussion

The metabolic syndrome and associated cardiovascular complications are a major health burden. There is limited information on how hyperglycaemia influences gene regulation. Because the liver plays a major role in energy homeostasis, we hypothesised that hepatocytes show robust gene expression changes in response to hyperglycaemia, some of which could be deleterious to cardiovascular health. Furthermore, we hypothesised that HDAC inhibition via VPA could reverse or attenuate some of these gene pathways.

We used high throughput RNA sequencing (RNA-seq) for its unbiased ability to detect expressed genes with greater sensitivity and accuracy than gene expression microarrays. With appropriate bioinformatics tools, regulatory events to genes and pathways (sets of genes) can be pinpointed in a way that is more efficient than single-gene assays. These tools were applied to identify genes and pathways that respond to hyperglycaemia and/or VPA in hepatocytes.

The major gene sets upregulated by hyperglycaemia were related to cholesterol metabolism, DNA replication and complement cascade and clotting cascades. The observation of elevated expression of clotting and complement factors is consistent with reports of these proteins being elevated in patients with diabetes. Interestingly, of these pathways, only complement and clotting cascades were attenuated by VPA.

The complement system is central to innate immunity against microorganisms and modulator of inflammatory processes; it comprises a complex and tightly regulated group of proteins involving various soluble and surface-bound components. Depending on the activation trigger, the complement cascade follows one of three pathways: classical, lectin or alternative^39^. Although these pathways differ in their mechanisms of target recognition, all converge in the activation of the central component C3. This process is followed by C5 cleavage and the assembly of the pore-like membrane attack complex, MAC. Important chemoattractants and inflammatory mediators are produced by the enzymatic cleavage of C3 and C5, which leads to the release of anaphylatoxins C3a and C5a^40^.

The coagulation system is another major blood-borne proteolytic cascade^41^. Upon activation of the coagulation cascade, a sequential series of serine protease-mediated cleavage events occur. Thrombin is activated from its zymogen prothrombin and then catalyze the polymerization of fibrin by cleaving small peptides from its subunits. This way, soluble fibrinogen is converted into insoluble fibrin, which allows the clot formation^42^. Thrombin also plays a key role in amplifying the cascade by feedback activation of coagulation factors^43^. Other components such as circulating red and white blood cells and platelets are incorporated into the clot structure. In addition, factor XIIIa, which is also activated by thrombin, provides further structural stability by cross-linking with fibrin^44^. In this context, weak clots are more susceptible to fibrinolysis and bleeding, while resistant clots may promote thrombosis^42^.

Coagulation and complement cascades share a common evolutionary origin^41^ and their interplay is highlighted by C3, C4, C5a and FB complement protein presence in thrombus^45^. Similarly, pro-coagulation enzymes thrombin and IXa, Xa, XI factors might activate complement cascade^46^. Moreover, MASP2, a component of lectin complement activation, is capable of cleaving coagulation factors prothrombin in thrombin, fibrinogen, factor XIII and thrombin-activatable fibrinolysis inhibitor in vitro^47,48^. Thus, understanding the crosstalk between these pathways has fundamental clinical implications in the context of diseases with an inflammatory and thrombotic pathogenesis, in which complement–coagulation interactions contribute to the development of complications^49^.

Liver, mainly hepatocytes, is responsible for the biosynthesis and secretion of the majority of complement and coagulation components. Furthermore, the promoter regions of these components are controlled by several common liver-specific transcription factors like HNFs and C/EBP^50^. Thomas and co-workers^51^ compared the genome-wide binding of Fxr and Hnf4α in mouse liver and characterized their cooperative activity on binding to and activating target gene transcription. Genes bound by Fxr and Hnf4α are enriched in complement and coagulation cascades, as well as in pathways related to drug metabolism. Furthermore, these transcription factors are involved in gluconeogenesis and glycogenolysis gene regulation^52,53^. Thus, a common transcription factor network may be controlling these immune and metabolic pathways.

The participation of complement in metabolism and metabolic disorders has recently received increasing scientific attention. Earlier studies demonstrate higher plasma C3 levels in diabetic patients compared to healthy individuals^6,54^. Increased complement gene expression has also been associated with adipocyte insulin resistance, waist circumference, and triglyceride levels^55,56^. MASP-1 and MASP-2 levels were significantly higher in children and adults with T1DM than in their respective control groups, whereas these proteins levels decreased when glycemic control improved^57^. In a recent study it was reported that in a murine model of diabetic nephropathy, genetic knock-out or pharmacological blockade of complement component 5a receptor 1 (*C5ar1*) conferred renal protection and attenuated disease-associated metabolic changes, further reinforcing the importance of the complement pathway^58^.

Metabolic syndrome, including diabetes mellitus, is associated with a procoagulant state, in which the clotting system is switched toward a prothrombotic state, involving reduced fibrinolysis, increased plasmatic coagulation, and platelet hyperactivity^43,59,60^. Intensive glycemic control with insulin reduces the impact of this procoagulant state by affecting components of clotting the system^60^. Abnormalities in the coagulation and fibrinolytic systems may contribute to the development of cardiovascular complications in patients with metabolic syndrome^43^ and consistent lowering of clotting factors are used for the treatment of acute cardiovascular syndromes^61^.

This study has limitations. The experimental results are derived from transformed HepG2 cells are informative but do not replace data derived from primary hepatic cells. Future work is proposed to examine the therapeutic benefit of VPA using pre-clinical models of transient and chronic hyperglycaemia^62,63^. While, HDAC inhibitors such as VPA are associated with changes in gene expression mediated in part by lysine acetylation of histone residues^64–70^ more recent studies have shown dramatic genome wide histone deacetylation associated with the transcription factors, CBP and EP300 using ChIP-Seq in primary vascular cells^23–25,71^. The experimental results presented in this article suggests lysine acetylation is associated with VPA attenuating *MASP* and *C3* gene expression we cannot rule out histone deacetylation of other complement genes. Furthermore, in addition to causing histone hyperacetylation, VPA has been shown to regulate replication-independent loss of DNA methylation^72^ that is consistent with elevated Tet2 DNA demethylase enzyme^73^. This seemingly extends the functional role of HDAC inhibitors such as VPA that alter lysine acetylation and deacetylation including DNA methylation by extracellular signalling mediated by hyperglycaemia^74^.

Several reports in the medical literature demonstrate that patients with neurological conditions taking VPA exhibit greater blood loss during surgery, impaired clotting, and reduced concentration of clotting factors^75–78^. Interestingly, emerging evidence points to complement cascade as having a causal role in some seizure types. Micro-array analysis identified complement cascade gene hyperactivation in brain tissue of epilepsy patients^79,80^. Studies in mice identified complement component C3 as necessary for acute viral infection associated seizures^81^. Our findings are consistent with these reports and indicate VPA mediated reduction of circulating complement and coagulation factors is a result of specific changes in hepatic gene expression. These changes in gene expression seem to be regulated at least in part by the relative abundance of H3K9ac at their promoter as observed here by ChIP qPCR analysis. As a prime initiator and important modulator of immunological and inflammatory processes, the complement system has emerged as an attractive target for early and upstream pharmacological intervention in inflammatory diseases^82^. In this context, repurposing clinically approved drugs such as VPA provides a time- and cost-effective alternative.

In conclusion, we could, for the first time, associate HDAC inhibition with complement and coagulation gene expression modulation. We demonstrate that coagulation and complement cascade genes were upregulated by hyperglycaemia and that these can be attenuated with VPA through its histone acetylation modulation ability. Future preclinical studies will resolve whether VPA can mitigate the complications of diabetes in vivo.

## Materials and methods

### Cell culture

HepG2 cells from ATCC at passage 9 were maintained in Dulbecco’s modified Eagle’s medium (DMEM) basal glucose (5.5 mM) (Gibco, Carlsbad, USA) supplemented with 10% fetal bovine serum (GE Healthcare, Chicago, USA) and penicillin and streptomycin (Gibco) (working dilution: 100 IU and 100 μg/mL, respectively). Cells were cultivated for 48 h in normoglycemic (LG 5.5 mM) or hyperglycaemic (HG) medium, containing D-glucose (Sigma, St. Louis, USA) up to a final concentration of 20 mM. Cells under LG and HG conditions were then treated with 1.0 mM VPA (Sigma) for another 12 h and compared with the respective untreated controls. The experiments were performed using conditions derived for VPA from previous studies by our group^64–70^.

### Transcriptome sequencing

Cells were disrupted with TRIzol (Qiagen, Hilde, Germany). RNA was isolated from TRIzol homogenates using the Direct-zol Kit (Zymo Research, Irvine, USA). RNA was quantified on Qubit (Thermo Fisher, Waltham, USA) and its quality was evaluated on the MultiNA bioanalyzer (Shimadzu, Kyoto, Japan). NEBNext Poly(A) mRNA Magnetic Isolation Module was used to enrich mRNA from 1 μg of total RNA. We used the NEBNext Ultra Directional RNA Library Prep Kit for Illumina (San Diego, USA) to generate barcoded libraries. Libraries were quantified on the MultiNA bioanalyzer (Shimadzu) and pooled to equimolar ratios for sequencing. Cluster generation was performed at a concentration of 10 pM (TruSeq SR Cluster Kit v3-cBot-HS) and the flow cell was run on Illumina HiSeq2500 generating 50 nt reads at AGRF Melbourne. Sequence data has been deposited to NCBI Gene Expression Omnibus under accession number GSE109140.

### Bioinformatics analysis

mRNA-seq data processing: Low quality bases (Qscore < 20) were removed from the 3′ end with FASTX Toolkit v0.0.13. Trimmed reads less than 20 nt were also discarded. The human genome sequence and annotation (GRCh37.75) set were downloaded from the Ensembl website (www.ensembl.org/info/data/ftp). Reads were aligned using STAR version 2.3.1p_r359^83^. Sorted bam files were generated with SamTools^84^ (version 0.1.19-44428cd). A gene expression count matrix was generated with featureCounts v1.4.2^85^ using a map quality threshold of 10. Genes with an average of fewer than 10 reads per sample were omitted from downstream analysis. EdgeR version 3.6.8 and limma version 3.20.9 were used to perform statistical analysis^86^. False discovery rate-controlled *p* values (FDR) ≤ 0.05 were considered significant. Gene expression of pathways was analyzed with GSEA-P using the classic mode^87^. A differential abundance score was obtained for each gene by dividing the sign of the fold change by the log10(*p* value). This score was used to rank genes from most up-regulated to most down-regulated as described previously^23^. Curated gene sets were downloaded from MSigDB^88^. To understand the correlation between effects of VPA and hyperglycaemia on global gene expression, we generated a rank–rank density plot of each detected gene. Genes were ranked as above and plotted using the filled contour plot function in R. Significance of two-dimensional enrichment of gene sets away from a uniform distribution was calculated Manova test of ranks in R as described previously^89^.

### Reverse transcriptase quantitative PCR

To validate some differentially expressed genes from the RNA-seq findings, we repeated the experiment using the same conditions of cell culture and treatment, isolated total RNA using the RNeasyMini Kit (Qiagen, Hilden, Germany) and prepared cDNA using the High-Capacity cDNA Reverse Transcription Kit (Applied Biosystems, Waltham, MA). Real-time PCR was performed using an Applied Biosystems 7500 Real Time PCR system following standard protocols and TaqManGene Expression assays (Applied Biosystems) for complement (*MASP2* (Hs00373722_m1), *C3* (Hs00163811_m1) genes. Target gene expression was normalized to the expression of *H3F3* (Hs02598544_g1) Relative quantification was achieved with the comparative 2−ΔΔCt method as described previously^90^.

### LI-COR Odyssey H3K9/14ac quantitation

Histones were isolated from HepG2 cells using the acid extraction method^91^. Proteins were separated using 4–12% gradient SDS-PAGE and transferred into a PVDF membranes (Immobilon-FL; Millipore). Blots were probed with primary antibodies specific for acetyl-histone H3 (06-599; Millipore), or total H3 (14269; Cell Signaling Technology) overnight at 4 °C. Following incubation with primary antibodies, membranes were rinsed and probed with appropriate mouse or rabbit secondary antibodies. Protein bands were visualized and quantified using Odyssey CLx image system (LI-COR Biotechnology).

### Chromatin immunoprecipitation (ChIP) qPCR

Chromatin immunoprecipitation was performed as previously described^16^. Three independent 10-cm plates of HepG2 cells growing under conditions above described (LG, LG VPA, HG, HG VPA) were used per immunoprecipitation. Cells were fixed with 1% formaldehyde in PBS for 10 min at room temp (RT) and the reaction quenched with glycine at a final concentration of 0.125 M. Sonicated chromatin was checked and anti-H3K9ac (C5B11 Rabbit mAb #9649, Cell Signaling Technology) enriched DNA was immunoprecipitated overnight. Soluble immunoprecipitated material was washed with a salt buffer sequence and collected. The eluted DNA was subjected to qPCR using specific primers (Integrated DNA Technologies) compared to inputs. The primers used in ChIP PCR are as follows; MASP2 forward ACACAGGTTCAGGCCACTTC, MASP2 reverse CACACCATGAGGTAGGTGGG; C3 forward GCTGAGGGAGGGGAAGTAGA, C3 reverse CCTGACCCTCCAAGAAGCAG.
